# Miscellanies

**Published:** 1838-01-01

**Authors:** 


### MISCELLANIES.

The following report arrived too late in the quarter for insertion in the Clinical department. Yet we are so anxious to encourage the Reports of the physicians and surgeons of hospitals, that we willingly give insertion to the present in this place. We would observe to those gentlemen, who are anxious to publish Clinical Reports in this Journal, that the Reports must be in the hands of the Editors by the middle of the current quarter. Suppose, for example, that a physician is anxious to have a report published on the 1st of January, 1838. He must transmit it to us by the 20th of November, 1837. Unless we receive the reports a month or six weeks prior to the day of publication, we are put to very great inconvenience.

### Surgical Report of some Cases in the County of Clare Infirmary. By George W. O'Brien, M.D. &c.

Case 1.—*Retention of Urine, from Contraction of the Orifice of the Urethra, consequent on Ulceration of the Penis.*

June 22, 1833.—Pat. Leonard, aged 28, contracted venereal about five years since; the ulceration carried away all the penis anterior to the scrotum. The orifice which remained has since been gradually closing, until it became not much larger than a pin-hole. About a year and a half ago he got retention of urine, from which he was relieved with much difficulty; and he was then directed to wear a short piece of a bougie, which he has latterly neglected. Since that period, the opening remained in a very contracted state, until the 20th of June, when it completely closed. When first seen, on the 22nd, he had not passed water for over 48 hours. He was then walking about the room nearly bent double, and in intense pain. He was covered by a cold clammy perspiration, which exhaled a strong urinous odour. His bladder was so distended, that it reached to the umbilicus. The orifice of the urethra could not be discerned, but a slight swelling appeared, just in front of the scrotum, communicating an obscure fluctuation to the finger.

A lancet was plunged into the tumor at the place where a cicatrix appeared, and where it was passed in for about an inch, the urine gushed out. An elastic gum catheter was then passed into the bladder, and about two quarts of urine, loaded with mucus, came away. The instrument was fixed in the urethra.

23rd. The catheter slipped out of the bladder last night. He was free from pain, but was obliged to make an effort to empty the bladder constantly, which he found much difficulty in doing. The instrument was replaced by a female gum catheter, which could be more easily fastened, owing to the shortness of the canal.

After a few days, he was able to dispense with the catheter, having recovered the power of emptying the bladder. A short piece of bougie was fixed in the orifice, and by gradually increasing its size, the opening was sufficiently dilated to prevent any inconvenience. It has not since contracted, nor has he found any difficulty in passing water.

Gradual contraction of the orifice of the urethra is a very unpleasant result of ulceration involving the corpus spongiosum—and a tendency to it, appears to prevail to a very remote period after the ulcer has healed. It rarely, I believe, proceeds to such an extent as completely to close the orifice, and require an incision for its relief.

Case 2. *Compound Fracture of the Elbow, with Laceration of the Brachial Artery and Median Nerve.*

Daniel M'Mahon, aged 43, a healthy countryman, admitted on the 24th of April, 1833.

On the evening before, whilst driving a car, it was upset, and fell on his left arm.

There is a contused and lacerated wound, about six inches long, in front of the elbow, extending from the inner condyle downwards to the outer side of the fore-arm. The radius and ulna are fractured near their upper extremities, and protruded through the skin to a considerable extent. The former is also separated from its connexion with the humerus.

All the muscles about the front and sides of the joint are severely contused and lacerated, and many of them hang outside the skin—they are of a very dark colour. The brachial artery and median nerve lie at the bottom of the wound, torn across, but the former had been secured with a ligature, immediately after the accident. The hand is in a state of extreme pronation; it is cold and quite insensible—it as well as the forearm are beginning to assume a livid hue. No pulsation can be felt at the wrist. There is a slight redness round the edges of the wounds—he lost a great deal of blood before assistance could be procured—he suffers intense pain. There are two smaller lacerations at the back of the elbow. Immediate amputation was proposed to him, but he refused to consent.

In about six hours afterwards, the pain having become almost insupportable, he agreed to its removal. Amputation was accordingly performed at the middle of the arm. During its performance considerable embarrassment was occasioned by his irresolution and restlessness. Three arteries required to be secured with ligatures.

He got a large opiate draught when removed to bed.

With the exception of severe night perspirations, which yielded quickly to the use of dilute sulphuric acid, no untoward circumstance afterwards occurred. The stump was completely healed in five weeks.

Injury of the principal vessels and nerves of a limb, complicated with fracture, is always one of the cases in which immediate amputation becomes necessary; the circulation and nervous influence being so impaired as to render gangrene of the parts below the injury an inevitable consequence. Even when the bone is uninjured, and the soft parts, including the artery and nerve, are the only textures which have suffered, the same result follows. An instance of this happened in the Infirmary, last year, where the brachial artery, its accompanying veins, and the median nerve, having been torn across, the bones being uninjured, gangrene seized on all the limb below the wound, and amputation became necessary.

Case 3.—*Fatal Injury of the Leg.* James Kerin, aged 20—a healthy countryman, brought into hospital at six o'clock on the evening of the 3rd of November, 1834.

About half an hour previously, whilst sitting on the side of a common car, had his right leg severely crushed between it and another, which was coming towards him at a rapid rate.

He is very pale, and cold; he suffers intense agony. Pulse 136, very small. There is an extensive wound at the upper and outer part of the leg, with great laceration of the subjacent parts. The tibialis anticus and peronei muscles are torn from their origins, and protruding. The upper end of the fibula is dislocated from its connexion to the tibia, and for four or five inches downwards is broken into several fragments. On introducing the finger into the wound, it passes readily backwards into the popliteal space, in which there is a large cavity, but no external wound. There is scarcely any hæmorrhage now, though there appears to have been a good deal before his arrival at the hospital. The wound was filled with dry lint, which was retained by adhesive straps, and covered with a roller, not tightly applied. A tourniquet was placed loosely round the thigh. He got 40 drops of laudanum and a drachm of compound spirit of ammonia. The latter ordered to be repeated in two hours, if the skin has not got warm.

12, p. m. Skin still universally cold. No appearance of reaction—a slight oozing of blood from the wound, which ceased on tightening the tourniquet.

To have a glass of warm wine and water every second hour.

Nov. 4th, 9 o'clock—No hæmorrhage since—pulse 150, thread-like. No return of heat to the surface—features contracted, and assuming a cadaverous aspect.

The wine and other stimulants were continued, but were quite unavailing. He died at two o'clock P. M.

*Dissection of the limb twenty hours after death.*—On making an incision into the ham, a large quantity of blood and air escaped. There is much extravasation amongst the adjacent muscles. The sciatic nerve, popliteal artery and vein are not injured; the muscles are torn from their origins, the anterior tibial artery is torn across. The upper part of the fibula having been dislocated, and comminuted, had evidently been driven with much force into the popliteal space.

CASE 4.—*Gun-shot Wound of the Popliteal Artery.* Mary Donahoe, æt. 20, admitted on the 27th of December 1834, at two o'clock P. M.

About 8 in the morning, received a wound by the accidental discharge of a gun loaded with shot—she was about ten yards from the muzzle of the piece when the shot was fired. The left thigh, about an inch and a half above the knee, was struck by the shot. About a hand's-breadth of the integuments at the inner part was perforated by shot-holes, the parts adjoining which, are discoloured from effusion of blood under the skin, and much contused. On attempting to stir the limb, crepitus could be felt, evidently produced by an extensively comminuted fracture of the thigh-bone. She was in a state of extreme collapse, her skin was universally cold, her face pale and her lips blanched —her pulse was quick and scarcely perceptible. She lost a large quantity of blood on her way into town, and there was still a good deal of it oozing through the shot-holes. There were also some grains lodged in the right thigh, but they lay immediately under the integuments. A tourniquet was put round the upper part of the thigh; a compress of lint applied to the wound and secured with a bandage, and she got a draught containing 30 drops of tincture of opium with a drachm of compound spirit of ammonia—warm applications to be kept constantly to her feet.

At three o'clock there was only a very slight discharge of blood from the wound. She was still cold, and her pulse extremely rapid. To get a mixture of warm wine with water and carbonate of ammonia for common drink. At five o'clock, some heat appeared to return to the surface, but her pulse remains the same—very little bleeding since; eight o'clock the appearance of re-action had not continued—pulse imperceptible — bleeding completely ceased—the injured limb quite cold; 11 P. M. died without a struggle—had been talking a few minutes before she expired.

*Dissection of the Limb.* The whole surface of the body was unusually pale and bloodless. On cutting into the wounded thigh, the whole muscles and cellular tissue were found infiltrated with blood. The bone was fractured about two inches above the condyles; the fractured ends were very much shattered and fissured upwards and downwards to a considerable extent. The knee-joint was uninjured; the popliteal artery and vein were wounded, and several grains of shot were driven far into the bone.

CASE 5. — *Wound of the Pharynx between the Os Hyoides and Thyroid Cartilage.* Matthew Nihill, aged 40, admitted at 11 o'clock on the morning of the 12th of February, 1835, having, about three hours previously, whilst in a state of mental depression, attempted to commit suicide by cutting his throat with a razor.

The wound extends transversely from the edge of the sterno-mastoid muscle on the right side to that of the left, dividing some of the anterior fibres of the latter.

He appears to have made two incisions, one passing between the os hyoides and thyroid cartilages, and the other detaching about an eighth of an inch of the upper part of the latter from the rest of its body—the separated portion hangs nearly loose in the wound. There is a considerable retraction of the edges of the wound, where the chin is raised. The incision has been carried to a considerable depth, and the pharynx and opening in the larynx are completely open to view; the epiglottis is uninjured, the incision having been made below its root. The left superior thyroid artery is cut across and bleeds profusely; the left carotid is laid bare to a considerable extent, and can be seen pulsating at the left extremity of the wound. He has lost a great deal of blood and is very much exhausted; the pulse is imperceptible and the surface cold and bloodless, but he is calm, and regrets the occurrence very much. He experiences much difficulty in articulating, but his voice is not rendered unintelligible. The hæmorrhage was completely arrested, by tying the thyroid artery. It was not deemed adviseable to employ sutures, as it would have been exceedingly difficult if not quite impossible to bring the cut edges of the wound together, on account of the manner in which the thyroid cartilage was wounded, besides the risk of the inflammation which would have been created by the ligatures, and the certainty of their being torn away by coughing or swallowing.

Some dry lint was gently laid into the wound, and an attempt was made to bring the edges as nearly in contact as possible, by having the head bent forward—he then got 30 drops of laudanum.

In the evening he was composed and quiet, and his breathing regular and easy—pulse 120, weak and intermitting.

13th.—Pulse 100, full and regular, breathing unembarrassed, countenance composed. Some milk and water was conveyed into his stomach by means of an elastic tube. Enema purgans.

14th.—Pulse 90, full, respiration natural, but there is a slight cough. The wound looks clean and well. Bowels free. Rept. enema.

18th.—The cough has completely ceased. Pulse 101, soft. Several detached pieces of the cartilage have come away. A purulent discharge is beginning to appear, and granulations are springing up all over the wound. Bowels regular, tongue clean. He is fed by means of the elastic tube.

22nd.—The wound has diminished a third, he can now swallow some soft bread with ease—to have broth. The exposed mucous membrane is not redder than natural.

27th.—Wound still contracting very much, he can now swallow almost any thing. Granulations looking healthy. Pulse natural. No cough. Appetite good.

On the 26th of April he was discharged from hospital, an opening about the size of a goose-quill remaining, his power of articulation and deglutition perfect.

The opening completely closed in about a month.

CASE 6. — *Poisoning by Sulphuric Acid.* J. Hall, 5 weeks old, admitted at eight o'clock on the evening of the 27th of December, 1834.

The woman who brought the child stated, that a few minutes previously, she detected its mother in the act of giving it vitriol mixed with panada, and the child had swallowed a good deal of it before she suspected what was doing. She immediately snatched the child from her and ran to the Infirmary.

The little creature was severely convulsed and writhed with agony immediately after swallowing it. When brought to the hospital it lay still and was insensible, and appeared at first to be dead.

The ordinary tube of a stomach-pump being much too large to pass, a gum-elastic catheter of a large size, was introduced into the stomach and fixed to a syringe. A lather of soap and tepid water was then injected and withdrawn repeatedly, and some pure water was then passed into the stomach until it returned quite free from acid. The child began to revive immediately, and in about five minutes after the instrument was withdrawn, commenced sucking the woman's breast.

On inspecting the inside of the mouth it as well as the dorsum of the tongue were found to be much excoriated. To get some carbonate of magnesia suspended in water immediately.

Some of the panada, which was kept by the woman, was then washed with distilled water, and the solution threw down a copious precipitate from muriate of barytes.

30th.—The child was griped occasionally during the night, but is much easier since morning. Its bowels have been moved several times. Mouth and tongue very sore. Ol. Ricini ℥ss.

31st.—Has had but little pain since, bowels moved several times.

January 3d, 1835.—Discharged, perfectly well.

---

### Dr. Dickson and Dr. Johnson.

Most of our readers have read the correspondence in the Lancet between these two gentlemen. Dr. Dickson appears to have taken mortal umbrage at an expression used by the Reviewer of his work in this Journal, where he merely asks the question if Dr. D. never *depletes* the pockets of his patients, seeing that the Doctor asserts that he never *depletes* at all. Any person of common good humour would have considered this as a mere joke; but Dr. Dickson seems to consider this insinuation as synonymous with the grave charge of being a PICK-POCKET. Now this could never have been intended by the Reviewer. Dr. D. boasts of having had *eight thousand private patients under his care in less than three years*; and allowing that he only received, on a moderate average, three fees from each patient, the worthy Doctor must have *pocketed* more than 24,000 pounds sterling in the short space of time above-mentioned!! This was a species of depletion which few physicians would be ashamed of—or unwilling to practise. Not so Dr. D. He singles out Dr. Johnson, without any evidence of his being the Reviewer, and loads him with abuse of all kinds—for all which Dr. Johnson freely forgives him. There was one charge, however, in Dr. Dickson's first letter in the Lancet, which Dr. J. could not treat with silence, because it involved an imputation of unprofessional conduct, namely, that of taking fees from his medical brethren for professional advice. Dr. D. publicly asserted that he possessed *proofs* of this misconduct in Dr. J. and Dr. Johnson denied the truth of the charge, and challenged Dr. Dickson to adduce his *proofs*. What did Dr. Dickson then do?

"Eager to wound, but still afraid to strike,"

he merely asks a question. Did Dr. J. or did he not, take some half-guinea fees from a Surgeon Gibbon?—modest assurance! It was as much as to say—"Pray my dear Doctor Johnson, just come forward with proofs of your own culpability, and it will save me a great deal of trouble." But Dr. J. was too old a sinner to make open confession before this impartial tribunal, and flatly denied the charge. The third attack was a brutum fulmen—a volley of general abuse—but a total abandonment of the accusation against Dr. Johnson, although he (Dr. D.) set out with the astounding asseveration that he had the proofs in his own hands!! In this humiliating situation, Dr. Johnson disdains to retort upon his adversary, though he has now the power and the opportunity to do so with effect. Dr. Johnson will only offer one piece of advice to Dr. Dickson—never again to make a charge of unprofessional conduct against any of his brethren, when he knows that he has not even the shadow of proof to substantiate it. In this attempt to affix a stigma on Dr. Johnson, he has affixed it on himself.

The following note from Dr. Stewart, of Portsmouth, will dispose of Dr. Dickson's interrogatory respecting "SURGEON GIBBON."

"Portsmouth, 13th Nov. 1837.

"Sir,—In the early part of 1835, I was requested to visit a gentleman just landed from an Indiaman, and who had taken up his abode at the Star and Garter Hotel in this town. On my arrival I was informed that it was a Dr.

Gibbon. I found him labouring under disease of the liver and general dropsy. After three or four days attendance, he was so far improved as to be able to proceed to London, which he was very anxious to reach, in order that he might consult his old friend, Dr. James Johnson, who had attended him during his previous visit to England. On his departure, he wished to remunerate me for my attendance, but I declined. His reply was—" *then you are as liberal as my good friend, Dr. Johnson, who never would take any reward from me for his advice.*" You are at liberty to make what use you please of this communication.

Yours truly,

To Dr. James Johnson. JAMES STEWART, M.D.

P.S.—Dr. Johnson has received nearly fifty letters from medical gentlemen, rebutting the accusation of Dr. Dickson, respecting fees from medical practitioners, and he takes this opportunity of thanking them all collectively for their kindness. It is obvious that, as only one instance was ventured upon by Dr. D. and that specific instance disposed of by Dr. Stewart's letter, it would be unnecessary to produce other documents bearing on the subject.

